# The Influence of Body Image, Insight, and Mental Health Confidence on Medication Adherence in Young Adult Women with Mental Disorders

**DOI:** 10.3390/ijerph18083866

**Published:** 2021-04-07

**Authors:** Eunmi Lee, Mi Heui Jang

**Affiliations:** College of Nursing Science, Kyung Hee University, Seoul 02447, Korea; shinhwa6em@naver.com

**Keywords:** mental disorder, body image, insight, medication adherence, women

## Abstract

The aim of this descriptive study was to investigate the impact of body image, insight, and mental health confidence on medication adherence among young adult women with mental disorders. Data collection occurred from August to September 2018. The study participants were 147 young adult women aged 19 to 45 with mental disorders who were psychiatry outpatients either getting treatment in general hospitals located in Seoul and Gyeonggi Province or receiving rehabilitation therapy through local mental health welfare centers in Korea, and agreed to participate in the study. The measurement tools used were the Body Image Scale; the Scale to Assess Unawareness of Mental Disorder, Korean short-form version; the Korean version of the Mental Health Confidence Scale; and the Korean version of the Medication Compliance Scale. The collected data were analyzed using descriptive statistics, *t*-test, analysis of variance, Pearson correlation analysis, and multiple regression analysis in SPSS/WIN 25.0 (IBM Corp., Armonk, NY, USA). Medication adherence among the study participants differed by age (F = 2.95, *p* = 0.042), religion (*t* = −2.06, *p* = 0.042), level of trust in psychiatrists (F = 5.40, *p* = 0.006), treatment duration (F = 4.48, *p* = 0.005), and noncompliance to medication regimens due to weight gain (*t* = −2.61, *p* = 0.010). Multiple regression analysis demonstrated that body image (β = −0.32, *p* < 0.001), insight (β = −0.24, *p* = 0.002), and mental health confidence (β = 0.24, *p* = 0.004) had a significant impact on the medication adherence of the participants. Body image, in particular, had the greatest influence on the medication adherence of the participants. This study found that body image, insight, and mental health confidence were important in improving medication adherence among young adult women with mental disorders. Practical, patient-centered, and individualized approaches that can improve medication adherence by seeking to understand negative perceptions regarding body image should be considered.

## 1. Introduction

Poor medication adherence is a major factor that influences recurrence among people with mental disorders, so supporting medication adherence is an important issue in the recovery and rehabilitation of patients with mental disorders [1]. In fact, 41–53% of patients with schizophrenia [2] and 20–60% of patients with bipolar disorder are reported to have poor adherence to medication [3]. Low medication adherence among people with mental disorders increases the frequency of recurrence and hospitalization and is strongly associated with an adverse course and prognosis, such as diminished quality of life, decreased job function, and an increased likelihood of attempting suicide [4].

According to previous studies, poor medication adherence among patients with mental disorders is affected by negative body image [5], low insight [6], and low self-efficacy [7]. It was found that patients with mental disorders did not adhere to their medication because antipsychotics contributed to an increase in body weight [8], and this trend was especially pronounced among female patients with mental disorders [5]. Atypical antipsychotic medications, which have recently become popular, show better results than typical antipsychotic medications regarding treatment effect, side effects, and medication adherence, but cause major metabolic disorders, including high blood pressure, impaired glucose tolerance, and abnormal lipid metabolism, and are more strongly associated with weight gain [9,10]. Regarding some antipsychotics, due to sex-specific differences in medication metabolism, female patients experience weight gain, an increased risk of diabetes or cardiovascular mortality, and side effects such as acne, halitosis, hirsutism, and other changes in appearance [11,12].

These side effects are not limited to female patients with mental disorders, but since women face widespread social pressure to attain an unrealistic standard of physical attractiveness, female patients with mental disorders may regard physical changes involving weight gain to be more important than male patients do [13]. In fact, female patients with mental disorders who considered themselves overweight or had a negative perception of their body image were found to be more likely not to take their medications [5]. Side effects of atypical antipsychotic medications have been frequently reported, but insufficient studies have examined the association between body image dissatisfaction and antipsychotic adherence among female patients with mental disorders.

An important factor that influences the medication adherence of people with mental disorders is insight, which refers to the self-recognition of one’s illness as well as the recognition that one has a mental disorder, treatment is necessary, and the mental disorder affects one’s social function [14]. Furthermore, 50–80% of patients with schizophrenia do not accept their illness or think they are just experiencing symptoms, so treatment adherence is low [15]. Few studies have examined sex differences in the association between insight and medication adherence, but given that females have been reported to have lower insight than males [16], there is a need to examine the association between insight and medication adherence among young adult women with mental disorders more closely.

In previous research on patients with mental disorders in the community, mental health confidence, a concept similar to self-efficacy, was reported to be an important factor that directly or indirectly influences treatment adherence among people with mental disorders [17,18]. Mental health confidence refers to patients’ sense of conviction or efficacy regarding their own behavior or decision-making in specific situations during the treatment or recovery process of mental disorders [19,20]. It was reported that patients with mental disorders who had low self-efficacy had poor medication adherence [7]. Self-efficacy is a major predictor of medication adherence [7] since self-efficacy has a positive influence on individuals’ cognitive regulation required for complex learning activities, such as acquiring knowledge about medications and problem-solving [21].

When mental disorders occur in early adulthood, the formation of intimacy and other important life decisions and experiences in this stage of life are delayed or missed, and the quality of life is diminished by repeated hospitalizations [22]. Therefore, to prevent recurrence and to increase the quality of life of young adult women with mental disorders, improving medication adherence is imperative [23]. Based on previous research [5,6,7], this study aimed to understand the impact of body image, insight, and mental health confidence on medication adherence among young adult women with mental disorders in South Korea.

## 2. Materials and Methods

### 2.1. Study Design and Participants

This study utilized a descriptive cross-sectional research design. The participants were patients with mental disorders either receiving outpatient treatment at two general hospitals in Seoul and Gyeonggi Province or using community mental health institutions (mental health welfare centers, day rehabilitation centers, and psychosocial rehabilitation centers) who voluntarily agreed to participate. Eligibility criteria were (1) young adult women aged 19 to 45 who were diagnosed with schizophrenia or bipolar disorder by a psychiatrist since the age of 19, the legal age of adulthood in South Korea, this age group having been chosen to include young adult participants; (2) those who did not have severe psychosis, as indicated by a Brief Psychiatric Rating Scale (BPRS) score lower than 36; and (3) those who were able to communicate and understand the questionnaire. The exclusion criteria were (1) those with neurocognitive disorders or addictive disorders and (2) those with severe psychosis, as indicated by a BPRS score higher than 37. The eligibility criteria were chosen to include participants who could answer questions requiring a subjective evaluation (e.g., about body image) to obtain insights into the relationships between medication adherence and other factors among young adult women who are maintaining a social life while being on medication in the community or as outpatients.

The sample size was calculated using the G*Power 3.1.9.2 program (Heinrich Heine University Düsseldorf, Düsseldorf, Germany). The required sample size for multivariate regression analysis with a significance level of 0.05, a medium effect size of 0.15, a power of 0.80, and 16 variables (13 sociodemographic variables, body image, insight, and mental health confidence) was 143. Considering a 20% dropout rate, 172 participants were included. Among the 172 participants who responded to the questionnaire, 147 were used for the final analysis, excluding 10 whose BPRS scores were greater than 37 and 15 whose answers were either insufficient or missing.

### 2.2. Measures

Body image was measured using the self-report Body Image Scale (BIS) developed and standardized by Kim and Park for patients with mental disorders [24]. The BIS is used to evaluate the degree of body image distortion in patients with mental disorders and consists of two subscales (physical appearance and physical health and strength). This scale is composed of 23 items measured on a 4-point Likert scale from “not at all” (0) to “very much so” (4). Items 4, 7, and 12, which were worded positively, were reverse-scored. The total score ranged from 0 to 92, with high total scores indicating highly negative attitudes toward their bodies. The original scale showed good internal consistency reliability, with a Cronbach’s alpha value of 0.80, and good construct validity with a two-factor structure explaining 31.4% of the variance in the study that introduced it [24]. The reliability of the scale in this study was shown by a Cronbach’s α value of 0.87.

Insight was measured using the Korean version of the Scale to Assess Unawareness of Mental Disorder (SUMD-K), originally developed by Amador et al. for patients with schizophrenia [25], translated by Song et al., and standardized for patients with schizophrenia [26]. The SUMD-K is a semistructured interview scale that consists of 3 items about overall insight (awareness of mental disorder, awareness of achieved effects of medication, and awareness of social consequences of mental illness) and 6 items eliciting information on symptoms. Although the scale was developed for patients with schizophrenia, the brief version is used with patients with bipolar disorder and mood disorders [25]. This scale is composed of 9 items measured on a 3-point Likert scale from “not applicable” (0) to “disagree” (3). The total score ranges from 0 to 27, with high total scores indicating low levels of insight. Amador et al. confirmed the reliability of the original scale with a Cronbach’s α of 0.94 [25], whereas Song et al. reported a Cronbach’s α of 0.71 [26]. The concurrent validity between the SUMD-K and the insight item of the Positive and Negative Syndrome Scale (PANSS) was high, with scores between 0.59 and 0.96 [26]. The reliability of the scale in this study was shown by a Cronbach’s α value of 0.70.

Mental health confidence was measured using the Korean version of the Mental Health Confidence Scale (MHCS-K), originally developed by Carpinello et al. for people with mental disorders [19], translated by Kwon [20], and standardized for people with mental disorders [20]. The MHCS-K consists of the following three subscales with 16 items: optimism (6 items), coping (6 items), and advocacy (4 items). Responses to this scale are given on a 5-point Likert scale from “not confident at all” (1) to “very confident” (5). The total score ranges from 0 to 80, with high total scores indicating higher mental health confidence. The reliability of the original scale was demonstrated by Carpinello et al., with a Cronbach’s α of 0.94 [19], and Kwon also reported a Cronbach’s α of 0.94 [20]. The MHCS-K showed good construct validity, with a three-factor structure explaining 62.3% of the variance in the study that introduced it [20]. The reliability of the scale in this study was shown by a Cronbach’s α value of 0.93.

Medication adherence was measured using the Korean version of the Medication Adherence Rating Scale (K-MARS), originally developed by Thompson et al. for people with mental disorders [27], translated by Jang et al., and standardized for patients with schizophrenia [28]. The K-MARS consists of 10 items on patients’ attitudes toward their medication, feelings after taking the medication, and whether patients do in fact take the medication. The 10 items of the K-MARS are divided into the following three subscales: subjective response (4 items), adherence behavior (4 items), and attitude toward medication (2 items). For each item, a score of 0 indicates nonadherence, and a score of 1 indicates adherence. The total score ranges from 0 to 10, with a higher score indicating better medication adherence. As a measure of the reliability of the scale in the original study by Thompson et al., Cronbach’s α was 0.75 [27], while Jang et al. reported Cronbach’s α to be 0.71 [28]. K-MARS showed good construct validity, with a three-factor structure explaining 53.4% of the variance in the study that introduced it [20]. In this study, the Kuder–Richardson formula 20 yielded a value of 0.73.

### 2.3. Data Collection

Data were collected from August to September 2018. After receiving approval for data collection from the patient’s primary physician in the department of psychiatry and from the administrators of the mental health center and daytime rehabilitation facilities, data were collected from the corresponding institutions. For each person who expressed interest in participating voluntarily in the study, the researchers or the designated staff at each facility distributed and explained the research protocol and obtained informed consent. The protocol stated that the collected data would not be used for purposes other than those set out in the form, that patients could withdraw from the study at any time, and that patient confidentiality would be ensured. Before completing the questionnaire, interviewers administered the BPRS and SUMD-K to participants who agreed to participate in the study in a space that only the researchers and participants could access. The interviewer then left, and the participant completed the questionnaire on her own. The completed questionnaire was then put in an envelope provided individually, sealed, and collected to maintain anonymity. A small cash reward was offered as compensation for their participation.

### 2.4. Statistical Analysis

Collected data were analyzed using SPSS version 25.0 statistical software. The general characteristics of the participants were analyzed using descriptive statistics, and the correlations between medication adherence and related factors were analyzed using Pearson’s correlation coefficients. In order to examine the factors influencing the degree of medication adherence, multiple regression analysis was used. Among general characteristics, variables that significantly influenced medication adherence were included in the regression model. Nominal variables were included as dummy variables for the analysis.

### 2.5. Ethical Considerations

The Institutional Review Board of H University (KHSIRB-18-045) and S Hospital (SCHUH 2018-08-002-001) in Seoul approved this study. The participants were informed that their participation in this study was voluntary and that they could withdraw at any time. The participants were also informed of the confidentiality of the data. The researchers obtained completed written consent forms from the study participants.

## 3. Results

### 3.1. General Characteristics and Disorder-Related Characteristics

The participants’ average age was 34.65 ± 8.20 years, and 47 participants (32.0%) were under the age of 30. Most participants (*n* = 124, 84.4%) were not married. The majority of the participants had graduated from high school (*n* = 79, 53.7%) and were religious (*n* = 94, 63.9%). Sixty-one participants (41.5%) reported a monthly household income of USD (United States Dollar) 862.5–2587.7, and 114 participants (77.6%) lived with family. The majority of the participants (*n* = 110, 74.8%) were diagnosed with schizophrenic spectrum disorder, and 37 participants were diagnosed with bipolar disorder (25.2%). Most participants (*n* = 103, 70.1%) responded that they trusted psychiatrists. The average duration of treatment was 9.18 ± 7.77 years. Thirty-one (21.1%) participants responded that they had never been hospitalized, and 42 (28.6%) responded they had been hospitalized four or more times. Thirty-seven (25.2%) participants responded that they did not know the antipsychotics they were currently taking very well. Most participants (*n* = 101, 68.7%) had experienced weight gain after taking antipsychotics, and 28 (27.7%) participants had stopped taking medication on their own due to weight gain. The average body mass index (BMI) was 25.23 kg/m^2^, and according to the standards of the Korean Society for Obesity, 50 (34.0%) participants were either normal weight (BMI < 23.0 kg/m^2^) or overweight (BMI = 23.0–24.9 kg/m^2^), 42 (28.6%) participants had obesity class I (BMI = 25.0–29.9 kg/m^2^), and 24 (16.3%) participants had obesity class II (BMI ≥ 30 kg/m^2^) (Table 1).

### 3.2. Descriptive Statistics of Body Image, Insight, and Mental Health Confidence

The score for body image among the participants was 41.80 ± 16.16 out of 92. The degree of insight was 16.29 ± 4.58 out of 27. The score for mental health confidence was 47.76 ± 14.52 out of 80, and that for medication adherence was 5.79 ± 2.59 out of 10 (Table 2).

### 3.3. Differences in Medication Adherence by General Characteristics and Disorder-Related Characteristics

The characteristics that were significantly related to differences in medication adherence among the participants were age (F = 2.95, *p* = 0.042), religion (*t* = −2.06, *p* = 0.041), level of trust toward psychiatrists (F = 5.40, *p* = 0.006), duration of treatment (F = 4.48, *p* = 0.005), and nonadherence to medication regimens due to weight gain (*t* = −2.61, *p* = 0.010). Participants aged between 30 and 39 had better medication adherence than those under 29. Religious participants had better medication adherence than those who were not religious. Participants who trusted their psychiatrists had better medication adherence than those who rated their trust as average. Better medication adherence was found among participants who had been treated for more than 20 years than among those who have been treated for 1–4 years. Among those who experienced weight gain after going on antipsychotics, those who responded they did not alter their medication on their own due to weight gain had better medication adherence than those who did (Table 1).

### 3.4. Correlations among Body Image, Insight, and Mental Health Confidence

Medication adherence had statistically significant negative correlations with body image (r = −0.616, *p* < 0.001) and insight (r = −0.397, *p* < 0.001), while it had a statistically significant positive correlation with mental health confidence (r = 0.565, *p* < 0.001) (Table 3).

### 3.5. Factors Influencing Medication Adherence of Participants

The tolerance limit (TL), which refers to the degree of correlations among independent variables of the regression analysis model, was greater than 0.1, and the variance inflation factor (VIF) was smaller than 10, indicating that variables were mutually independent and multicollinearity was not an issue (TL: 0.557–0.904, VIF: 1.106–1.796) (Table 4).

Multivariate regression analysis was conducted with medication adherence as the dependent variable and continuous variables of body image, insight, and mental health confidence that influenced medication adherence and categorical variables of age, religion, level of trust in psychiatrists, and duration of treatment. Religion and level of trust in psychiatrists were coded as dummy variables. Nonadherence to medication due to weight gain was only answered by 101 participants who experienced weight gain and thus was excluded from the list of independent variables. The final regression model included age, religion, level of trust in psychiatrists, duration of treatment, body image, insight, and mental health confidence. The regression model explained 42.9% of the variance (adjusted *R*^2^ = 39.2%). Body image was the strongest predictive variable (β = −0.32, *p* < 0.001), followed in order by insight (β = −0.24, *p* = 0.002) and mental health confidence (β = 0.24, *p* = 0.004) (Table 4).

## 4. Discussion

Medication adherence among young adult women with mental disorders in this study was on average 5.79 out of 10, which is moderate. The score is lower than what was reported in other studies that used the same tool, such as 6.95 in the study by Jang et al. [28]. A reason for the lower medication adherence in this study might be that other studies included participants of both sexes, in light of previous reports that women had lower medication adherence than men [23]. Although not displayed in a table, body image was more negative among participants aged 19 to 29 years than those in their 30s and 40s (F = 4.75, *p* = 0.010). Not only did participants who had been treated for 1–4 years have a more negative body image (F = 6.48, *p* < 0.001), but they also had lower medication adherence than participants who had been treated for more than 5 years or for more than 10 years. This result indicates that medication adherence is lower in participants younger than 30 or those who are young and early in their diagnosis, with a treatment duration of less than 4 years. The incidence rate of mental disorders in early adulthood is steadily increasing, and the resulting social cost is very large [29]. The early stages of mental disorders such as schizophrenia coincide with a transitional phase in life when individuals prepare to enter adulthood, experiencing many challenges, changes, and adaptations [30]. It is very important to facilitate an early diagnosis and early intervention for mental disorders at this stage to prevent the disorder from becoming chronic, to promote recovery from symptoms, and to complete developmental challenges in adulthood [31]. Early intervention services, which are grounded in the principle of intervening at the earliest possible time, encompass assertive case management with relatively low caseloads, pharmacotherapy, and multimodal psychosocial interventions, enable effective delivery of care for people experiencing early psychosis [32]. These services facilitate engagement with treatment and promote accessibility; furthermore, early interventions have been found to diminish symptom severity, to decrease the relapse rate, and even to reduce hospital admissions [32]. However, mental health programs in South Korea are focused on the rehabilitation of chronic mental disorder patients, rather than on the early treatment of newly diagnosed patients or those who are in the early stages of the disorder [33]. Therefore, the results of this study indicate that to increase medication adherence among young adult women with mental disorders, the focus should be on medication adherence of patients younger than 30 or those diagnosed within the past 5 years.

The findings that body image was more negative and medication adherence was lower among women under 30 than women in other age groups can be explained as follows. According to a previous study [34], women in their 20s tend to observe and worry about how their bodies look more than women in other age groups, and care the most about their appearance according to the eyes of others. This finding is identical to the results of other previous studies with people with mental disorders [5]. In this study, medication adherence was more strongly correlated with body image than with insight or mental health confidence, and a more negative body image was associated with lower medication adherence. This finding indicates that body image plays an important role in medication adherence among young adult women with mental disorders. Therefore, mental health experts should consider negative body image, especially among young female patients with mental disorders under the age of 30, and provide patient-centered and useful intervention strategies so that patients’ psychological demands related to body image are reflected and medication adherence can be maintained.

Participants who highly trusted psychiatrists had higher medication adherence than those who did not trust psychiatrists. This result is in line with those of other previous studies of patients with schizophrenia or bipolar disorder [35,36]. Participants adhered to medication better when they perceived psychiatrists as having a cooperative relationship with them and as providing empathy and support [37], so developing trust between participants and psychiatrists is very important. The chronic nature of schizophrenia requires ongoing engagement between clients and treatment team members. Psychiatric nurses are in a pivotal position to educate clients about the use of shared decision-making tools for use in their partnership with the prescribing clinician [38]. Participants who responded that they stopped taking their medication after experiencing weight gain due to antipsychotics had lower medication adherence than participants who did not. Previous studies also found that weight gain interfered with medication adherence [8]. Weight gain is associated with a high risk of cardiovascular diseases or type II diabetes [14], and obesity can affect patients’ prognosis by damaging their confidence, diminishing their social function, and causing other social side effects [39,40]. Therefore, mental health nurses should monitor side effects of antipsychotics, focusing on weight gain and metabolic disorders among young adult women with mental disorders who are prescribed antipsychotics. When necessary, nursing intervention programs that can improve negative body image, such as weight management programs and cognitive behavioral therapy, should be applied.

As expected, in the regression analysis, which yielded the main findings of this study, a more negative body image, lower insight, and lower mental health confidence were associated with poorer medication adherence. Body image, insight, and mental health confidence explained 39.2% of the variance in medication adherence in the regression model. An especially interesting result is that body image more strongly predicted medication adherence than insight or mental health confidence. This result supports previous studies that found poor medication adherence when women with mental disorders considered themselves overweight or perceived their body image negatively, were not satisfied with their body types, and wished they were thinner [5], and studies reporting that medication adherence was more strongly associated with subjective stress from weight than objective weight indicated by BMI [34,41]. Therefore, in order to improve medication adherence among young adult women with mental disorders, a patient-centered approach to factors interfering with medication adherence is necessary, including close assessments of perceptions of and dissatisfaction with body image and efforts to understand negative or distorted beliefs and attitudes toward body image dissatisfaction. If body image distortion or dissatisfaction is very severe among young adult women with mental disorders, a cognitive behavioral therapy approach is needed. If body image dissatisfaction is related to the side effects of antipsychotics, a change in medication might also be necessary.

Previous studies [37,41] reported that insight among people with mental disorders had a close correlation with improvements in medication adherence. Therefore, implementing programs that enhance insight among people with mental disorders to improve medication adherence is important. Mental health confidence, which was also found to be a factor that improved medication adherence, refers to patients’ sense of conviction or efficacy regarding their own behavior or decision-making in specific situations during the treatment or recovery process of mental disorders [18,19]. Self-efficacy is an important factor related to medication adherence [7] since self-efficacy has a positive influence on individuals’ cognitive regulation required for complex learning activities, such as acquiring knowledge about medications and problem-solving [21]. The results of this study support those of a previous study of community members with mental disorders [16], which reported that self-efficacy was an important factor that influenced treatment compliance directly and indirectly. For patients with schizophrenia who need antipsychotic maintenance therapy both in the early stages of the disorder and in the long term, voluntary medication uptake is more important than temporary supervision of medication administration, and it is important to increase mental health confidence through self-care strategies to improve medication adherence [42]. Medication side effects and pressure, as well as coercion from healthcare providers and family, can reduce patients’ motivation to take antipsychotic medication [37,41]. Therefore, steps should be taken to seek ways to increase mental health confidence so that participants can adhere to medication according to their own autonomous judgment and behavior based on a supportive and cooperative alliance of the family and treatment team [41].

The limitations of this study are as follows. The first is the issue of selection bias. The participants in this study were young adult women with mental disorders in a selected area, and it is difficult to generalize the results. Second, the average duration of treatment among the study participants was 9.18 years, which indicates that they were chronic patients. It is difficult to generalize the results of this study to all patients with mental disorders. Therefore, future studies should focus on young adult women with mental disorders who were newly diagnosed or diagnosed in the past 5 years. Third, some important variables related to medication adherence, such as medication side effects and types of antipsychotics, were not included in this study. Additional studies including these variables are necessary.

## 5. Conclusions

Young adult women with mental disorders who participated in this study had moderate levels of medication adherence, and poorer medication adherence was found among participants who were younger than 30, who had never been hospitalized, whose treatment duration was under 5 years, who had experience stopping medication due to weight gain from antipsychotics, and who did not have trust in psychiatrists. Medication adherence was better when participants’ insight and mental health confidence were high, and medication adherence was especially low when perceptions of body image were more negative (rather than insight or mental health confidence). The results of this study indicate that mental health care providers should evaluate whether nonadherence among young adult women is related to their body image, assess the patients’ body image, and provide a tailored approach in order to increase adherence. Therefore, mental health care providers need to consider the variables that showed meaningful relationships in this study in order to help promote medication adherence among participants.

## Figures and Tables

**Table 1:** Differences in medication adherence by general characteristics (*n* = 147).

Variables	Categories	*n* (%)	Medication Adherence		
			M ± SD	*t*/F	*p*
Age (years) M (SD) = 34.65 ± 8.20	<30	47 (32.0)	5.02 ± 2.88	3.24	0.042
	30–39	52 (35.4)	6.27 ± 2.40		
	40–44	48 (32.6)	6.02 ± 2.37		
Marital status	Single	124 (84.4)	5.78 ± 2.62	0.28	0.754
	Married	9 (6.1)	6.33 ± 2.92		
	Divorced and others	14 (9.5)	5.50 ± 2.28		
Education	Middle school or less	16 (10.9)	6.63 ± 1.96	1.21	0.302
	High school	79 (53.7)	5.82 ± 2.63		
	University or higher	52 (35.4)	5.48 ± 2.70		
Religion	Yes	94 (63.9)	6.12 ± 2.54		
	No	53 (36.1)	5.21 ± 2.62	−2.06	0.041
Monthly income (US dollars)	<862.4	29 (19.7)	5.90 ± 2.27	1.24	0.297
	862.5–2587.6	61 (41.5)	6.20 ± 2.29		
	2587.7–4315.8	26 (17.7)	5.42 ± 2.63		
	≥4315.9	31 (21.1)	5.19 ± 3.29		
Living condition	Alone	33 (22.4)	5.88 ± 2.31	0.25	0.804
	With family	114 (77.6)	5.75 ± 2.68		
Diagnosis	Schizophrenic spectrum disorder	110 (74.8)	5.95 ± 2.48	1.26	0.209
	Bipolar disorder	37 (25.2)	5.32 ± 2.90		
Level of trust in psychiatrists	High	103 (70.1)	6.21 ± 2.38	5.40	0.006
	Moderate	30 (20.4)	4.53 ± 2.99		
	Low	14 (9.5)	5.36 ± 2.41		
Treatment duration (years)	1–4	58 (39.5)	4.86 ± 2.83	4.48	0.005
	5–9	30 (20.4)	6.33 ± 2.15		
	10–19	38 (25.9)	6.29 ± 2.40		
	≥20	21 (14.2)	6.67 ± 2.15		
Number of past hospitalizations	None	31 (21.1)	5.32 ± 3.07	0.95	0.440
	1	37 (25.2)	5.49 ± 2.79		
	2	21 (14.3)	5.62 ± 2.25		
	3	16 (10.9)	6.31 ± 2.12		
	≥4	42 (28.5)	6.29 ± 2.35		
Level of knowledge of medication	High	54 (36.7)	5.44 ± 2.71	1.19	0.307
	Moderate	56 (38.1)	5.79 ± 2.22		
	Low	37 (25.2)	6.30 ± 2.91		
Experience of weight gain after taking antipsychotics	Yes	101 (68.7)	5.75 ± 2.72	−0.25	0.801
	No	46 (31.3)	5.87 ± 2.32		
Nonadherence to medication regimens due to weight gain	Yes	28 (27.7)	4.64 ± 2.74	−2.61	0.010
	No	73 (72.3)	6.18 ± 2.61		
Body mass index, kg/m^2^	<23.0	50 (34.0)	5.68 ± 2.61	0.27	0.847
	23.0–24.9	31 (21.1)	6.03 ± 2.48		
	25.0–29.9	42 (28.6)	5.60 ± 2.76		
	≥30	24 (16.3)	6.04 ± 2.51		

M = mean; SD = standard deviation.

**Table 2:** Degrees of body image, insight, mental health confidence, and medication adherence (*n* = 147).

Variable	Number of Items	M ± SD	Minimum	Maximum	Range
Body image	23	41.80 ± 16.16	15	86	0–92
Insight	9	16.29 ± 4.58	7	27	0–27
Mental health confidence	16	47.76 ± 14.52	17	79	16–80
Medication adherence	10	5.79 ± 2.59	0	10	0–10

M = mean; SD = standard deviation.

**Table 3:** Correlations among body image, insight, mental health confidence, and medication adherence (*n* = 147).

Variable	Body Image	Insight	Mental Health Confidence
Insight	0.266 (<0.001)		
Mental health confidence	−0.603 (<0.001)	−0.248 (0.003)	
Medication adherence	−0.616 (<0.001)	−0.397 (0.001)	0.565 (0.001)

**Table 4:** Factors influencing medication adherence (*n* = 147).

Variables	B	SE	β	*t*	*p*
Constant	7.60	1.68		4.51	0.001
Age (years)	0.01	0.03	0.02	0.22	0.828
Religion	0.09	0.37	0.02	0.24	0.807
Level of trust in psychiatrists					
Moderate	−0.23	0.46	−0.04	−0.51	0.612
Low	−0.25	0.56	−0.03	−0.44	0.658
Treatment duration (years)	0.02	0.03	0.07	0.81	0.420
Body image	−1.15	0.31	−0.32	−3.72	0.001
Insight	−1.15	0.36	−0.24	−3.19	0.002
Mental health confidence	0.67	0.23	0.24	2.89	0.004

B = regression coefficient; SE = standard error; β = standardized beta; dummy variable: religion (reference = no); levels of trust in psychiatrists (reference = high); adjusted *R*^2^ = 0.392; F (*p*) = 11.66 (<0.001).

## Data Availability

The data presented in this study are available from the authors upon reasonable request.
